# Piezoelectric nanofiber/polymer composite membrane for noise harvesting and active acoustic wave detection†

**DOI:** 10.1039/c9na00484j

**Published:** 2019-10-28

**Authors:** Nuanyang Cui, Xiaofeng Jia, Anan Lin, Jinmei Liu, Suo Bai, Lu Zhang, Yong Qin, Rusen Yang, Feng Zhou, Yongqing Li

**Affiliations:** Institute of Nanoscience and Nanotechnology, Lanzhou University Gansu 730000 China qinyong@lzu.edu.cn; School of Advanced Materials and Nanotechnology, Xidian University 710071 China rsyang@xidian.edu.cn; State Key Laboratory of Solid Lubrication, Lanzhou Institute of Chemical Physics, Chinese Academy of Sciences Lanzhou 730000 China; College of Naval Architecture and Ocean Engineering, Naval University of Engineering Wuhan 430033 China liyongqing@126.com

## Abstract

Being one of the most common forms of energy existing in the ambient environment, acoustic waves have a great potential to be an energy source. However, the effective energy conversion of an acoustic wave is a great challenge due to its low energy density and broad bandwidth. In this work, we developed a new piezoelectric nanogenerator (PENG), which is mainly composed of a piece of piezoelectric nanofiber/polymer composite membrane. As an energy harvester, the PENG can effectively scavenge a broad low-frequency (from 50 Hz to 400 Hz) acoustic energy from the ambient environment, and it can even scavenge a very weak acoustic energy with a minimum pressure of only 0.18 Pa. When a drum was used as an excitation source, the maximum open-circuit voltage and short-circuit current density of the PENG reached 1.8 V and 1.67 mA m^−2^, respectively. In addition, the PENG had a good stability and its output frequency and amplitude were closely related to the driving sound wave, which made the PENG capable of detecting acoustic signals in the living environment and have the potential to be applied as a self-powered active acoustic detector.

## Abstract 2

The PENG exhibited a good performance for harvesting and detecting low-frequency acoustic energy with a minimum sound pressure of 0.18 Pa.

## Graphical abstract

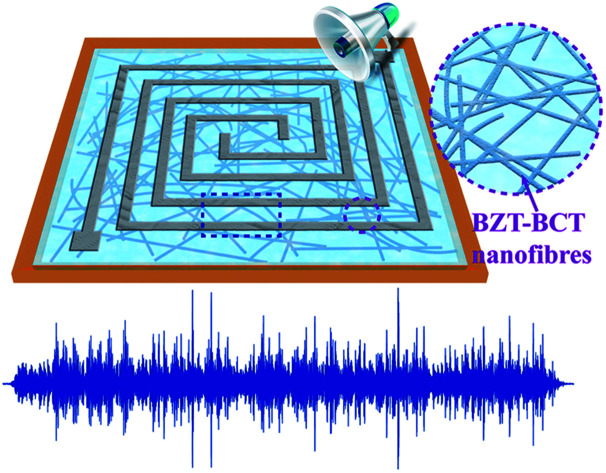

## Introduction

Acoustic waves are one of the most common energy forms existing in the environment. If acoustic energy could be effectively harvested, it has the potential to power millions of widely-distributed sensor network in internet of things. However, its low energy density and broad bandwidth make acoustic energy difficult to harvest. There are three main mechanisms that have been utilized to fabricate sound wave energy harvesters: electromagnetic induction,^1^ piezoelectric effect,^2–6^ and electrostatic effect.^7–11^ An electromagnetic acoustic wave generator has a high sensitivity and output performance, but the complicated and expensive manufacturing technique make it not suitable for large-scale and dispersive applications. The other two kinds of acoustic energy harvesters are the piezoelectric nanogenerator (PENG) and triboelectric generator (TENG). Energy conversion in a TENG is achieved by coupling the triboelectric effect and the electrostatic effect.^12^ Though a relative higher output has been reported, the performance of the TENG was largely determined by the charge density, which must be high enough for a normal output through long accumulation time and is easily dissipated by moisture, dust, and intermittently driven force.^13–15^ Therefore, it is difficult for TENGs to achieve their best performance in a real working environment. Compared with TENG, the working mechanism for the PENG is based on the piezoelectric property of piezoelectric materials. When the PENG is deformed by external forces, a piezoelectric potential is created,^16^ which is not susceptible to the environment. In addition, our previous work demonstrated the PENG's ability to convert mechanical energy into electric energy and its sensitivity to tiny forces.^17^ Consequently, its performance is barely affected by the environment, which makes PENGs a promising candidate for outdoor applications to harvest sound energy. To realize the harvesting of acoustic energy, the PENG must meet the following requirements. First, it should be very sensitive to tiny forces, which means that its response to sound pressure should be fast and accurate. Second, it should be able to harvest the noises of broadband frequency around our living environment. Third, its working frequency should be regulated to adapt to different situations.

Among previous research, the piezoelectric effect was mostly focused on acoustic sensing rather than energy harvesting and utilization, where the accurate voltage response of the acoustic signals represented by the intensity and frequency is of a greater concern.^18–20^ However, when paying attention to the effective harvesting of acoustic energy, a higher output energy and conversion efficiency are highly desired, especially the output current.^21^ Although the sound pressure created by acoustic wave is generally weak, greater deformation along the tension direction of a flexible membrane is possible to increase the output current. On the other hand, lead zirconate titanate (PZT) is widely used in the production of acoustic harvesting devices due to its compatibility with nano/micro-fabrication techniques and its easy development using a sol–gel process.^5,22^ Unfortunately, PZT has also aroused intensive concerns for human health and the environment.^23,24^ Therefore, it is important to explore lead-free piezoelectric membrane. 0.5Ba(Zr_0.2_Ti_0.8_)O_3_–0.5(Ba_0.7_Ca_0.3_)TiO_3_ (BZT–BCT) nanofiber is a lead-free piezoelectric ceramic with a high piezoelectric coefficient (620 pC N^−1^),^25–28^ which has been reported as a biocompatible PENG.^29^ However, they have not been explored as an acoustic energy harvester until now.

In this paper, we demonstrate a membrane PENG device, in which piezoelectric BZT–BCT nanofibers serve as the energy conversion material. Polydimethylsiloxane (PDMS) and BZT–BCT nanofibers were assembled into a piece of membrane. This composite nanofiber membrane showed an ability to harvest energy from ambient noise. The membrane PENG responded to acoustic waves ranging from 50 Hz to 400 Hz as well as produced an open-circuit voltage of 1.8 V and a peak output current density of 1.67 mA m^−2^. The minimum pressure response was only 0.18 Pa. Furthermore, the restoration of the driving sound signals *via* the output current implied that the device has the potential to be used as an active acoustic detector.

## Results and discussion

### BZT–BCT nanofiber piezoelectric membrane

**Fig. 1:**
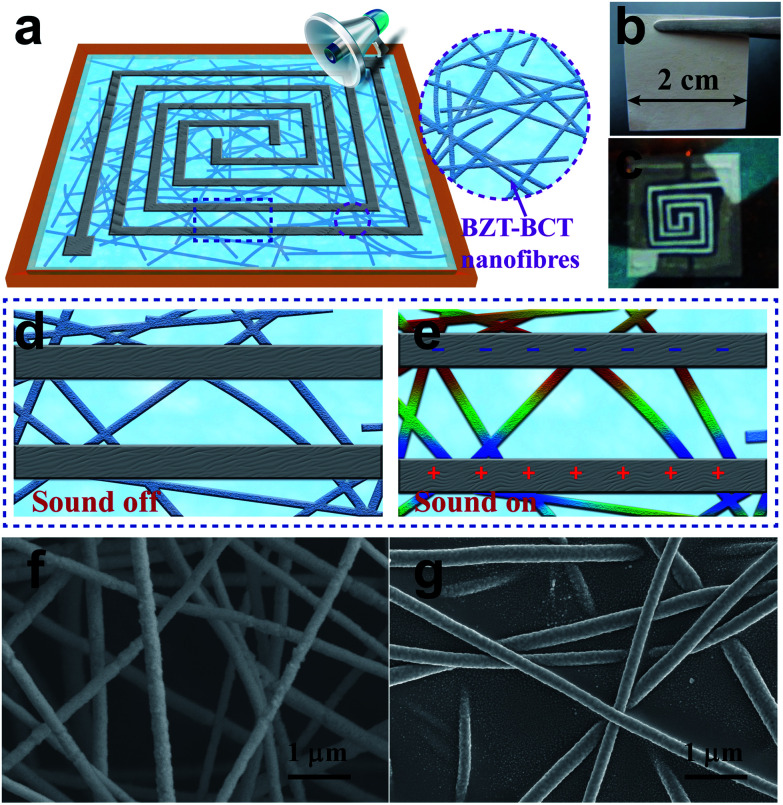
Morphology and structure of the PENG. (a) Schematic illustration of the PENG. (b and c) Optical photographs of the BZT–BCT membrane and BZT–BCT membrane with electrodes in the PENG. (d and e) Working mechanism of the PENG. (f and g) SEM images of the BZT–BCT nanofibers and a BZT–BCT/PDMS nanofiber composite membrane.

In our work, the PENG was made into a monolayer membrane structure as shown in Fig. 1a. Herein, the BZT–BCT nanofibers were used to fabricate the monolayer membrane structure. Fig. 1b shows an optical photograph of a piece of the BZT–BCT vibrating membrane with a side length of 2 cm and thickness of only 5 μm. The spiral silver electrode was deposited on the BZT–BCT membrane through magnetron sputtering using a shadow mask (Fig. 1c). The detailed fabrication process is depicted in the Experimental section. The nature frequency of the square membrane with its four sides fixed can be expressed as follows:1
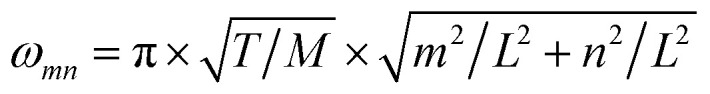
where *T* is the uniform surface tension per unit length, *M* is the surface mass density, *m* and *n* are arbitrary integers, and *L* is the side length of the membrane. The compound parameter, 
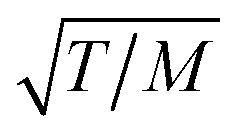
, refers to the wave propagation velocity in the membrane. Eqn (1) shows that the membrane could be induced resonance at many frequencies with different values of *m* and *n*. In addition, the nature frequency could be easily adjusted by changing the parameters of the membrane, such as the surface tension *T*, the surface mass density *M* or the side length *L*. This adjustment enabled our devices to work in a wider frequency range and improved its adaptability for harvesting noise energy from different environments. The working mechanism of the PENG is shown in Fig. 1d and e. When the acoustic source was set near the PENG and turned on, the BZT–BCT membrane vibrated along the vertical direction of the membrane surface. Consequently, the strain in the membrane changed periodically, radially, symmetrically, and isotropically in all directions. Therefore, the BZT–BCT nanofibers between the two electrodes were stretched and recovered alternately with the changing strain. During this process, the electrons in the external circuit flowed continuously between the two electrodes to respond to the change in the piezoelectric potential. The detailed working mechanism is depicted in (Fig. S1†). Fig. 1f shows the microstructure of the sintered BZT–BCT textile, where the BZT–BCT nanofibers were distributed randomly with a diameter of about 200 nm. The BZT–BCT nanofibers were prepared *via* an electrospinning method as discussed in a previous report.^30^ The detailed preparation process for the BZT–BCT nanofiber textile is depicted in the Experimental section. The BZT–BCT textile was fabricated into a thin vibrating membrane with almost no loss of its thin and light features by filling with PDMS which provided the textile with more support to prevent the tearing of the nanofibers. The detailed preparation process for the BZT–BCT vibrating membrane is depicted in the Experimental section. Fig. 1g shows a Scanning Electron Microscopy (SEM) image of the BZT–BCT vibrating membrane, which demonstrates that the PDMS filled in the gap of the BZT–BCT textile and formed a reinforced concrete structure together with BZT–BCT nanofibers. This structure not only enhanced the tensile strength of the membrane but also kept the thin and light features of the BZT–BCT textile.

### Performance of the membrane PENG for noise energy harvesting and active acoustic wave detection

**Fig. 2:**
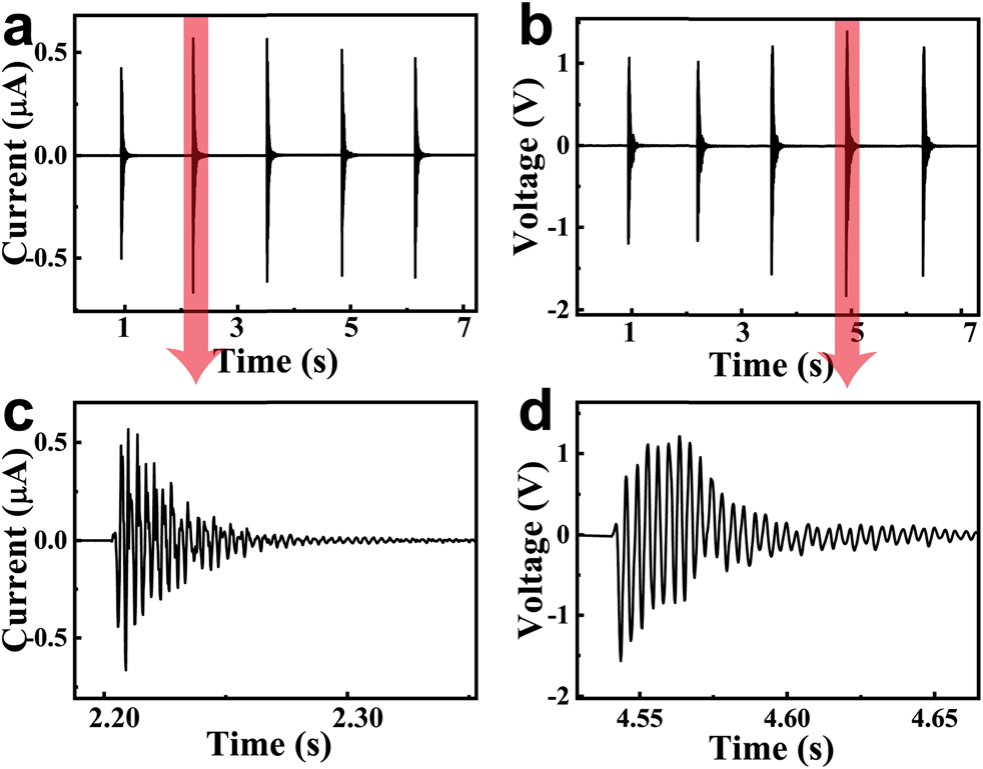
(a and b) The output voltage/current of a PENG excited by drumbeats. Each wave envelope corresponds to one beat of a drumstick. The device was placed 2 cm above the drum. The maximum output voltage and current of the PENG reached 1.8 V and 0.67 μA, respectively. (c and d) The details for an output voltage/current wave envelope, respectively.

Fig. 2 shows the output current and voltage of the PENG when a drum was used as an excitation source. The PENG was fixed on an iron support that was about 2 cm above the drum head. When we beat the drum head with a drumstick, the vibration of the drum spread through the air to the PENG. Fig. 2a and b show five current/voltage wave envelopes corresponding to five beats on the drum head. The maximum output current and voltage of this PENG reached 0.67 μA and 1.8 V, respectively (the current signal and voltage signal were measured through a Stanford Research System SR560 low-noise current preamplifier and SR570 low-noise voltage preamplifier. Their input resistances are 100 Ω and 100 MΩ, respectively.). The whole driven process was completely manual. The knocking force cannot be controlled the same. Therefore, for all of the beats, there was a slight deviation between the peak values for the output current/voltage. Fig. 2c and d show the details for the output current/voltage wave envelope. These damped waves reflect the damped vibration on the drum head. It was found that the frequency of the output electric signal was about 300 Hz, which was in accordance with the natural frequency of the drum.

**Fig. 3:**
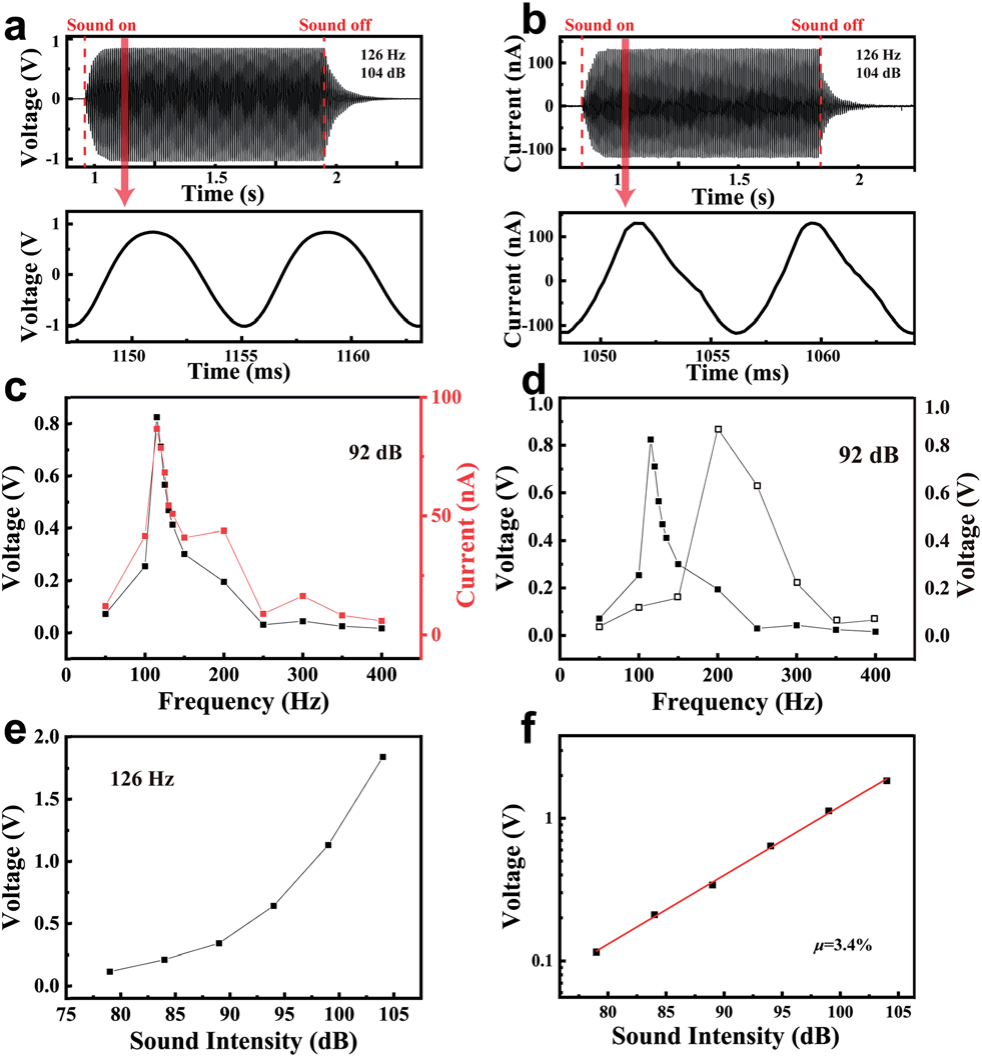
(a and b) The output voltage/current of a PENG driven by a sound of 126 Hz and 104 dB. The details for the output voltage/current wave are marked by the red line. (c) The output voltage/current of a PENG driven by an acoustic source with different frequencies (the loudness was kept at 92 dB). (d) The output voltage of two different devices with different membrane tension. (e) The output voltage of the PENG in different environments (the frequency was kept at 126 Hz). (f) The relationship between the peak voltage and sound intensity in semi-logarithmic coordinates.

In order to further investigate the performance of the PENG, including the frequency and intensity response characteristics, a mini speaker was applied as the excitation source, which sent out sounds with different frequencies controlled by a LabVIEW program. This measurement system generated a sine acoustic wave with a frequency ranging from 50 Hz to 400 Hz. The sound intensity was monitored by a noise meter. In the testing process, the PENG was fixed above the speaker and kept a certain distance from the speaker. First, the generator was driven under the sound of 126 Hz and 104 dB for one second. Fig. 3a and b show that the peak value of the output current and voltage reached 132 nA and 1.0 V, respectively. The PENG device also exhibited a very fast response speed, which reached a stable output within only 0.08 seconds. For comparison, a TENG often takes several minutes to achieve a relatively stable output.^15^ When the driving sound stopped, the vibration membrane gradually stabilized after 0.22 seconds of damping vibration. As shown in the enlarged view, the frequency of the voltage/current curves was 125.6 Hz (the acoustic wave was 126 Hz). The voltage curve was similar to the sinusoidal form. However, the current curve was seriously deformed, which was chiefly due to the smaller resistance of the current preamplifier leading to a faster current change.

The generator was driven at a series of frequencies ranging from 50 Hz to 400 Hz while keeping the sound intensity at 92 dB. The corresponding peak values for the voltage and current are shown in Fig. 3c. In this test, the output signal changed with the frequency of the driving acoustic wave and there was an explicit relationship between the output signal and the driving frequency, which was used as a criterion to detect the acoustic wave. In this frequency range, the maximum output voltage and current were 0.45 V and 44 nA at 115 Hz, respectively. The minimum output voltage and current were 9 mV and 3 nA at 400 Hz, respectively. Therefore, the resonant frequency of this device was 115 Hz and the PENG showed a certain selectivity to the frequency of the acoustic wave. In order to improve the detection performance of the PENG, its resonant frequency was adjusted to a value close to the working frequency in certain circumstances. As shown in eqn (1), if we increased the tension degree *T*, the resonant frequency of the PENG moved to higher values. For example, when the BZT–BCT membrane's tension was increased in the device, the resonant frequency increased accordingly to 200 Hz as shown in Fig. 3d. Fig. 3e shows the peak value of the voltage signals of the PENG driven by 126 Hz acoustic waves with the sound intensity increasing from 79 dB to 104 dB. The output signals increased monotonically. In fact, a simple expression between the voltage peak and sound intensity (SIL) was obtained (the derivations for eqn (2) and the energy conversion efficiency are shown in Derivation S1 and Derivation S2, respectively, in the ESI†):

2 log_10_ *V* = 0.05 × SIL + 0.5 × (log_10_ *k* + log_10_ *RI*_0_)

where *V* is the peak value of voltage, SIL is the sound intensity, SIL = 10 × log_10_ *I*/*I*_0_, *I* is the energy flux density of the sound, *I*_0_ is a basic parameter that equals to 10^−12^ W m^−2^, *R* is the load resistance, which equals to 100 MΩ, and *k* is a scale factor that is related to the energy conversion efficiency. Eqn (2) shows that the logarithm of the voltage is linearly proportional to the SIL. After taking the logarithm of the voltage peak in Fig. 3e, the result is shown in Fig. 3f. These data points show a strict linear relationship with the slope of the fitting being 0.049 and the intercept of the fitting being −4.76. By the fitting intercept, the scale factor *k* was calculated as 0.05. Furthermore, the energy conversion efficiency *μ* was also calculated as 0.86% for our device. It is worth mentioning that the sound pressure dropped exponentially from 3.17 Pa to 0.18 Pa when the sound intensity decreased from 104 dB to 79 dB. The low sound pressure (0.18 Pa) caused the PENG's response at the sound intensity of 79 dB. The output current and voltage were 10 nA and 70 mV, respectively.

**Fig. 4:**
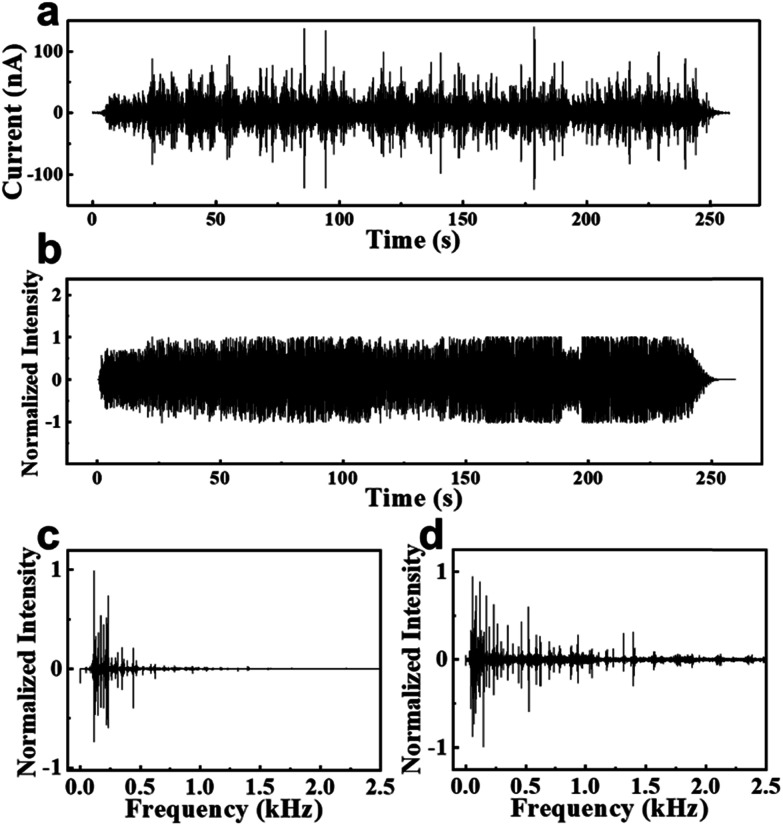
(a) Current responses of a PENG to the sound of a Chinese popular song, “*Su Xiu*”. (b) The original waveform of this popular song. (c and d) The corresponding frequency spectra for (a) and (b), respectively.

Considering the sensitivity to low sound pressure, the response bandwidth feature, and the adjustability of a membrane PENG, it should be suitable to harvest ambient noise energy. From Fig. S2,† the PENG successfully harvested noise at different environments such as a noisy workshop, a helicopter taking off, an alarm, and a man's voice, and converted them into electricity. Fig. 4a and b show the current responses and the original waveform of a PENG to a Chinese popular song, “*Su Xiu*”, respectively. Their corresponding frequency spectra are shown in Fig. 4c and d, respectively. It can be seen that the current response of the PENG exhibited some distortion in the waveform compared with the original waveform, which was mainly caused by its response defects to the high-frequency signals. This suggested that an improved design was needed. However, after converting the current signals in Fig. 4a back to sound signals (Video S1†) using a MATLAB program, the melody and lyrics were still easily distinguishable and similar to the original song. These results implied the potential value of the PENG as an active acoustic wave detector.

**Fig. 5:**
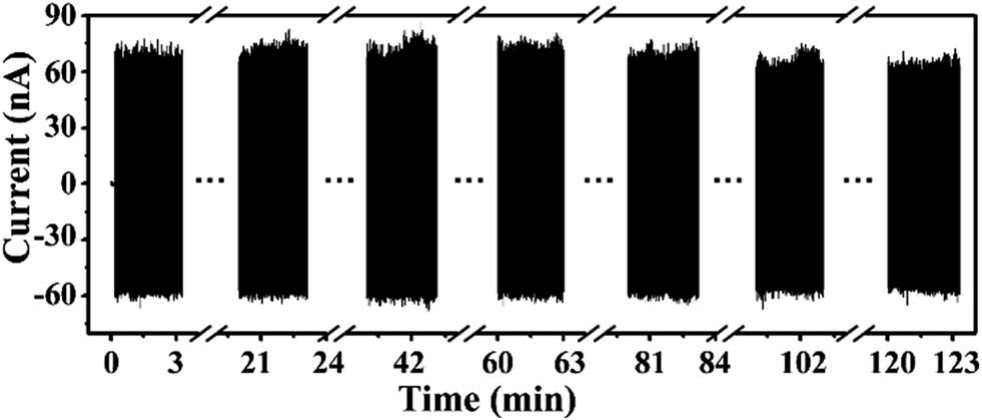
The electrical output of a PENG working for more than 2 hours at a frequency of 120 Hz. (Since the amount of data was too large at a sampling rate of 5000, the data were only acquired for 3 minutes every 20 minutes).

Moreover, benefiting from the reinforced composite structure of the BZT–BCT membrane, the PENG had a great mechanical robustness and long-time working ability. Fig. 5 shows the stability of the PENG driven by a 120 Hz and 95 dB acoustic wave. The PENG almost exhibited no decay in output even after working continuously for over 2 hours.

## Methods

### Preparation of the electrospun BZT–BCT nanofiber textile

During the electrospinning process, a piece of aluminum foil was used to collect the BZT–BCT precursor nanofibers. Then, these BZT–BCT precursor nanofibers were peeled off from the aluminum foil and put on an alumina ceramic substrate to volatilize the organic solvent under an atmospheric environment. Finally, the precursor textile was sintered in a muffle furnace at 850 °C for 3 hours at a ramp rate of 2 °C per minute.

### Preparation of the BZT–BCT/PDMS nanofiber composite membrane

The photoresist layers were fabricated by spin-coating on two clean PET films with a rotation speed of 3000 rpm and baking for 2 min at 90 °C on a hot plate. Herein, the photoresist layer was used as a sacrifice layer, which could be dissolved in an acetone solution. After the sintered BZT–BCT textile was lightly placed on one PET film, dilute PDMS (curing agent : PDMS : diluter = 1 : 10 : 100) was added dropwise on the BZT–BCT textile to infiltrate it. Then, the mixture was spin-coated on the PET film with a rotation speed of 1000 rpm for 1 minute. The BZT–BCT textile was covered with another PET film to form a sandwich structure. Later, the sandwich structure was put on the hot plate at 90 °C with a heavy object keeping it close to the hot plate. After the curing of the PDMS was complete, the whole sandwich structure was immersed in the acetone solution to detach the BZT–BCT membrane from the PET films.

### Fabrication of the membrane-based PENG for noise harvesting

First, a square hollow (1.8 × 1.8 cm^2^) was cut in the middle of a piece of PET film (8 × 8 cm^2^). Then, a layer of the photoresist was spin-coated on the PET film with a rotation speed of 3000 rpm and baked for 2 min at 90 °C on a hot plate to cure the photoresist. Herein, the photoresist layer was used as a sacrifice layer that was dissolved in the acetone solution. A PDMS layer was then spin-coated on the photoresist layer with a rotation speed of 1000 rpm. The whole PET film stood on a horizontal table for 1 hour. Later, a 2 × 2 cm^2^ square of the BZT–BCT nanofiber membrane was placed over the hollow part of the PET film. During this process, the edge of the membrane needed to be infiltrated in the uncured PDMS layer. After the PDMS layer was solidified at 90 °C for 30 min, the square BZT–BCT membrane and the PDMS layer were attached together to form a piece of complete elastic square film (8 × 8 cm^2^ with no hollow sections). Then, the whole part was immersed in acetone solution to dissolve the photoresist sacrifice layer and detach the PDMS film from the PET film. The entire film (PDMS film and the BZT–BCT membrane) was stretched and covered on a square annular support, similar to covering a drum. Afterwards, a group of spiral silver electrodes were deposited on the BZT–BCT membrane through magnetron sputtering using a shadow mask (Fig. 1d). The gap between the two electrodes was about 0.5 mm. Finally, this device was polarized in an electric field of 1 kV mm^−1^ at 100 °C for 1 hour.

## Conclusions

In summary, we develop a piezoelectric nanofiber/polymer composite membrane-based PENG for harvesting sound wave energy from ambient environments. The lowest sound intensity for driving the PENG is 79 dB which corresponds to a sound pressure of only 0.18 Pa. The generator has a wideband characteristic, which can respond to sound waves ranging from 50 Hz to 400 Hz. Meanwhile, by adjusting the resonant frequency of the device *via* tension control, the PENG adapts well to different environments. Owing to its high sensitivity and broad bandwidth characters, the newly designed PENG can convert the output current signal back to the sound signal, which implies its potential to be an active acoustic detector for sound distinguishing and recording.

## Conflicts of interest

There are no conflicts to declare.

## Supplementary Material

NA-001-C9NA00484J-s001

NA-001-C9NA00484J-s002
