# Supplementary material for: Piezoelectric nanofiber/polymer composite membrane for noise harvesting and active acoustic wave detection
Source: Nanoscale Adv. 2019 Oct 28;1(12):4909–14. doi: 10.1039/c9na00484j (PMC9416852; doi:10.1039/c9na00484j)
Supplement: NA-001-C9NA00484J-s001 [file NA-001-C9NA00484J-s001.pdf]

Electronic Supplementary Material (ESI) for Nanoscale Advances.
This journal is © The Royal Society of Chemistry 2019

Supplementary information

Piezoelectric Nanofiber/Polymer Composite Membrane for Noise Harvesting and Active Acoustic Wave Detection

Nuanyang Cui‡[a,b], Xiaofeng Jia‡[a], Anan Lin[a], Jinmei Liu[a], Suo Bai[a], Lu Zhang[a], Yong Qin[a,], Rusen Yang[b,*], Feng Zhou[c] and Yongqing Li[d,*]*

[a]Institute of Nanoscience and Nanotechnology, Lanzhou University, Gansu 730000, China

[b]School of Advanced Materials and Nanotechnology, Xidian University, 710071, China

[c]State Key Laboratory of Solid Lubrication, Lanzhou Institute of Chemical Physics, Chinese Academy of Sciences, Lanzhou, 730000 China

[d]College of Naval Architecture and Ocean Engineering, Naval University of Engineering, Wuhan 430033, China

*Corresponding author email: qinyong@lzu.edu.cn

rsyang@xidian.edu.cn

liyongqing@126.com

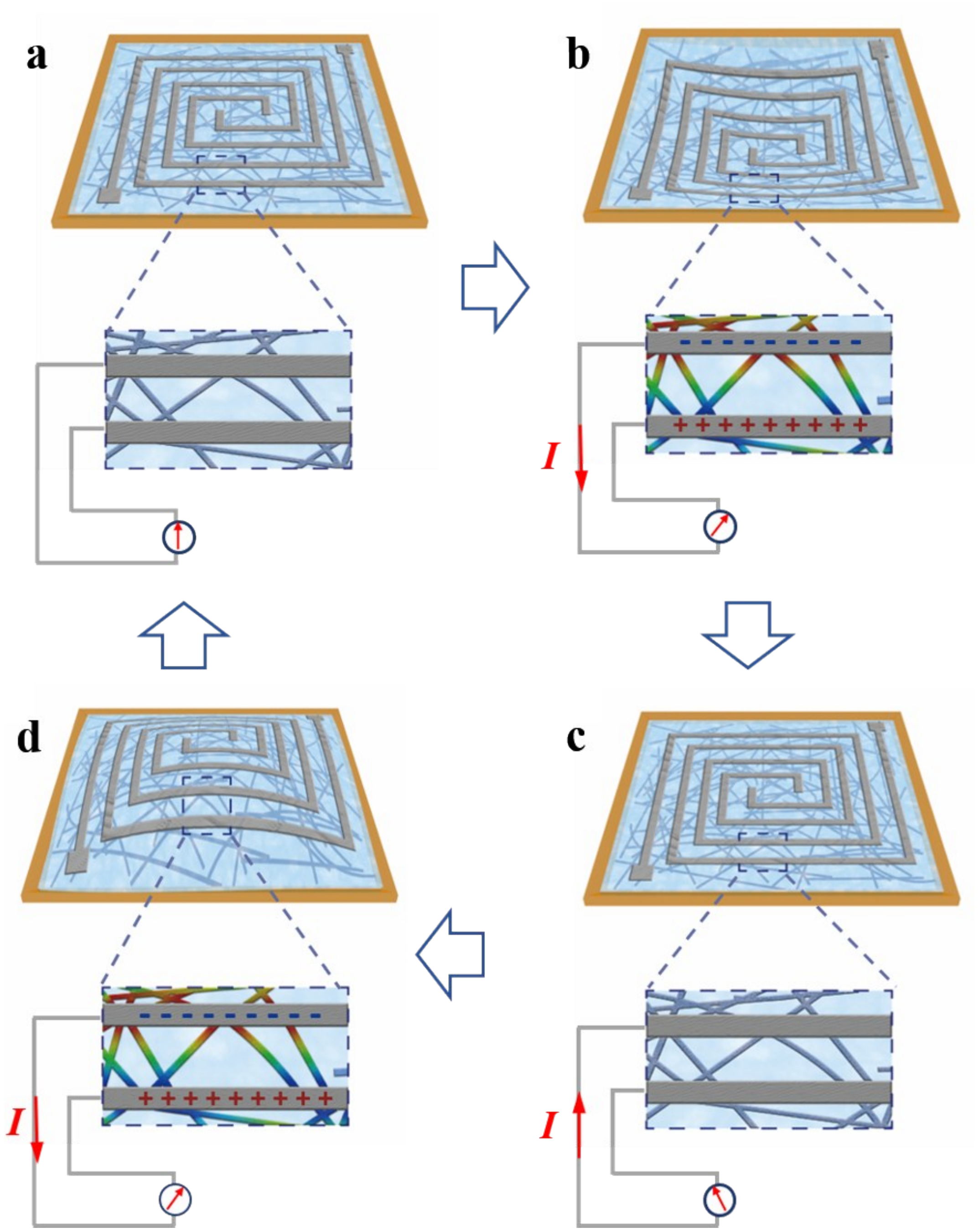

Figure S1. The detailed working mechanism of the membrane PENG: (a) the original state of the PENG; (b) the process of the membrane bent downward, where the membrane and nanofibers are stretched and result in a piezoelectric potential, and the external electrons flow to screen the piezoelectric potential and thus form a current; (c) the situation of the membrane recovered, where the piezoelectric potential disappears and the external electrons flow reversely to the original state and form a reverse

current; (d) the process of the membrane bent upward, where the membrane and nanofibers are still stretched and result in a piezoelectric potential similar to (b).

Derivation S1. The derivation process of equation 2.

$$\frac{V^2}{R} = kI$$

$$\frac{V^2}{RI_0} = k\frac{I}{I_0}$$

$$log_{10}\frac{V^2}{RI_0} = log_{10}k\frac{I}{I_0}$$

$$log_{10}V^2 - log_{10}RI_0 = log_{10}k + log_{10}\frac{I}{I_0}$$

$$2log_{10}V - log_{10}RI_0 = 0.1SIL + log_{10}k$$

$$log_{10}V = \frac{1}{20}SIL + \frac{1}{2}(log_{10}k + log_{10}RI_0)$$

V is the peak to peak value of voltage, *SIL* is the sound intensity, $SIL = 10log_{10}I/I_0$, *I* is the energy flux density of sound, I_0 is the basic parameter which equals to 10^{-12} Wm^{-2}, *R* is the load resistance which equals to 100 MΩ, and *k* is a scale factor which is related to the energy conversion efficiency.

Derivation S2. The derivation process of the energy exchange efficiency μ.

First, we can get the intercept of the fitting is -4.76,

and $\frac{1}{2}(log_{10}k + log_{10}RI_0) =- 4.76487$

$$k = 2.953E - 6.$$

The voltage curve follows a sine function, so

$$P_{power} = \frac{\left(\frac{(V/2)}{\sqrt{2}}\right)^2}{RS} = \mu I$$

$$\frac{V^2}{R} = 8 \times S\mu I$$

so

$$k = 8 \times S\mu.$$

S is the active area of the vibrating membrane which equals to 0.43E-4 m^2. So the energy exchange efficiency μ equals to 0.86 %.

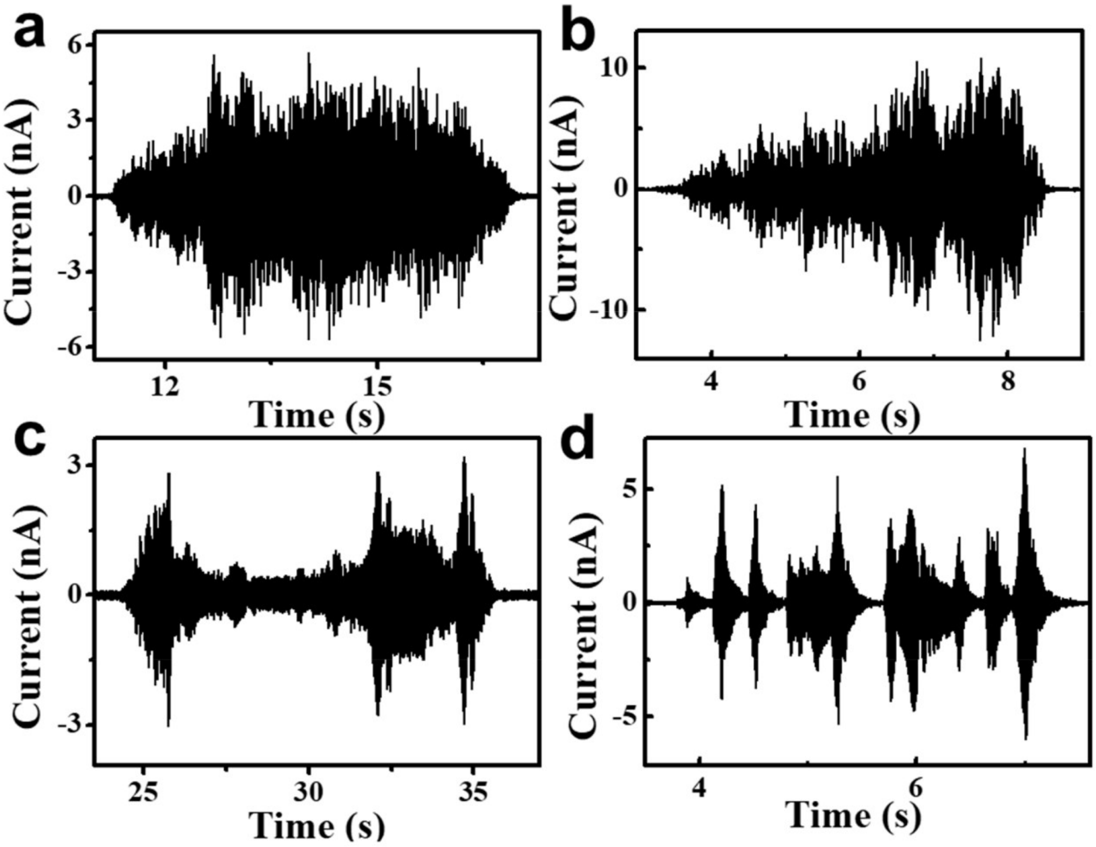

Figure S2. Current responses of PENG to (a) the noise of a simulative workshop; (b) the sound of a helicopter taking off; (c) the sound of an alarm; (d) a man's voice saying "Merry Christmas!"

Video S1. A restored song converted from the output current signals of the PENG.
